# Factors related to spiritual health in Chinese haemodialysis patients: A multicentre cross‐sectional study

**DOI:** 10.1002/nop2.535

**Published:** 2020-05-31

**Authors:** Yingjun Zhang, Guifang Xue, Yunlan Chen, KeRun An, Lin Chen

**Affiliations:** ^1^ Hemodialysis Center Department of Nephrology West China Hospital of Sichuan University Chengdu China; ^2^ Hemodialysis Center The Second People’s Hospital of Liangshan Yi Autonomous Prefecture Liangshan Yi Autonomous Prefecture China; ^3^ Hemodialysis Center Department of Nephrology The First People’s Hospital of Liangshan Yi Autonomous Prefecture Liangshan Yi Autonomous Prefecture China

**Keywords:** acceptance of illness, family function, haemodialysis, hope, spiritual health

## Abstract

**Aim:**

This study aimed to investigate the current situation of the spiritual health of maintenance haemodialysis (MHD) patients in China and analyse the influencing factors.

**Methods:**

A total of 418 patients who underwent maintenance haemodialysis in three grade A tertiary hospitals were selected. The influencing factors were evaluated with demographic questionnaire, the Functional Assessment of Chronic Illness Therapy‐Spiritual Well‐Being (FACIT‐Sp‐12), Family APGAR Index, Herth Hope Index (HHI) and Acceptance of Illness Scale (AIS).

**Results:**

Spiritual health was positively correlated with the HHI, Family APGAR and AIS scores. Nationality, HHI score, Family APGAR score and AIS score were independent influencing factors of spiritual health. MHD patients had a moderate level of spiritual health. Nationality, hope, family function and acceptance of illness were significant predictors of spiritual health. Patients who have higher hope levels, better family functioning and better illness acceptance may maintain better spiritual health.

## 1 INTRODUCTION

Chronic kidney disease (CKD) is a syndrome where the structure, function or both of kidneys continuously change, affecting the health of the individual. In recent years, CKD has become an important public health problem in many countries. The increasing prevalence rate, the low awareness rate, the low intervention rate, the occurrence of complications and the high treatment cost have greatly affected quality of life and increased the social and national economic burden (Dehbashi, Sabzevari, & Tirgari, 2015; Shdaifat & Manaf, 2012). With the progression of CKD, an increasing number of patients enter end‐stage renal disease (ESRD). The number of patients with ESRD is expected to increase to 45,000 by 2030 (SH, 2014). A Chinese study showed that approximately 19.5 million of 100 million CKD patients developed ESRD (Zhang et al., 2012). Haemodialysis is an effective method for treating ESRD, and more than 93.00% of dialysis patients choose maintenance haemodialysis (MHD); this rate increases by 18.70% annually (Prasad & Jha, 2015). MHD can extend the life of ESRD patients appropriately and improve renal function; however, it is still an invasive treatment and needs to be carried out for the rest of a patient's lifetime. However, it cannot completely replace kidney function and patients must still go back and forth to the hospital constantly, which is one of its limitations. Moreover, patients often experience a variety of physical and psychological symptoms and poor quality of life in the haemodialysis treatment process (Abdel‐Kader, Unruh, & Weisbord, 2009; Cukor, Cohen, Peterson, & Kimmel, 2007; Jahromi, Hosseini, Razeghi, Pasha Meysamie, & Sadrzadeh, 2010; Jhamb, Weisbord, Steel, & Unruh, 2008).

## 2 BACKGROUND

With the progress of medicine, the connotation of health has been gradually enriched to include not only physical health, mental health and social health but also spiritual health. Spiritual health is a subjective, abstract and complex concept. It is considered the ability to discover and understand one's basic purpose in life; learn to experience love, happiness, peace and contentment; and help oneself to achieve one's full potential when facing the problems and stress caused by disease (Dehbashi et al., 2015). Spiritual health enables people to have strong faith and hope to constantly transcend adversity and achieve their life goals. Spiritual health is the same for everyone; it does not apply only to religious people but is also related to each person's subjective will (Yang, Yen, & Chen, 2010). The WHO added spiritual health as an important part of health, pointing out that the overall health of patients in body, mind, society and spirit should be emphasized and improved (Dhar, Chaturvedi, & Nandan, 2011). Thus, spiritual health is increasingly valued by medical staff. Studies have found that spiritual health promotes health in all dimensions and positively affects quality of life (Hammermeister & Peterson, 2001; Wallace & Forman, 1998).

Spiritual health has become an important factor in predicting the quality of life of haemodialysis patients (Alshraifeen et al., 2020). Haemodialysis treatment changes the daily life of patients by requiring a specific diet associated with fluid restrictions and low potassium intake; in addition, the arteriovenous fistula and haemodialysis catheter change the appearance of patients, which may affect their level of hope (Ottaviani et al., 2014). Higher spirituality scores have been found to be correlated with lower symptomatic pain, higher levels of hope, better mental health and greater satisfaction with life (Tanyi & Werner, 2008). Although relevant studies have been performed in other countries, the factors associated with spiritual health remain poorly understood in Chinese MHD patients. Little is known about how spiritual health affects the course of illness. Therefore, it is important to determine the factors influencing spiritual health and meet patients’ spiritual needs to improve their spiritual health and quality of life. Due to differences in living environment and economic and social conditions, the present study aimed to highlight the factors of spiritual health in MHD patients from multiple haemodialysis centres in mainland China.

## 3 METHODS

### 3.1 Sample

This study was a multicentre cross‐sectional survey and the sample comprised 418 MHD patients at three hospitals in Sichuan, China, who were enrolled from October 2019–November 2019. Participants strictly matched the following criteria for selection: (a) age ≥18 years; (b) duration of maintenance haemodialysis of more than 3 months; (c) MHD treatment 2‒3 times per week; (d) normal speech, literacy and comprehension skills; and (e) informed consent and voluntary participation. The exclusion criteria included (a) inpatient MHD; (b) cognitive or behavioural impairment; (c) serious complications or other serious diseases and inability to take care of oneself; and (d) refusal to participate.

### 3.2 Data collection

Investigators were uniformly trained by the researchers. Before the investigation, patients were informed that the study was conducted under the principles of anonymity and confidentiality and the patients voluntarily signed the informed consent form. In addition, the researcher introduced the purpose and significance of the study to the patients. The questionnaires were distributed through the Questionnaire Star app, and the researchers instructed the patients to scan the QR code and complete the survey while undergoing MHD in the haemodialysis centre. For patients without smartphones, the researchers administered and collected the survey on the spot.

### 3.3 Instruments

The demographic questionnaire was designed by the researcher according to the research objectives and collected information such as age, gender, nationality, marital status, education level, type of medical insurance and religious belief. The Functional Assessment of Chronic Illness Therapy‐Spiritual Well‐Being (FACIT‐Sp‐12) consists of 3 dimensions (12 items) and uses a 5‐point Likert scale ranging from 0–4 (from very inconsistent to very consistent) (Canada, Murphy, Fitchett, Peterman, & Schover, 2008). The total score ranges from 0–48, and the higher the score is, the better the patient's spiritual health is. A score <24 indicates a low level of spiritual health, 24–35 a medium level of spiritual health and ≥36 a high level of spiritual health. Cronbach's alpha coefficient in this study was 0.921.

The Family APGAR Index was designed by Smilkstein (1978) and is used to test individuals' satisfaction with their family functioning. There are five items in this scale; the possible responses are "usually" (2 points), "sometimes" (1 point) and "almost never" (0 points). The total score is between 0–10; 7–10 indicates a functional family, 4–6 indicates a moderately dysfunctional family, and 0–3 indicates a severely dysfunctional family. The retest reliability of the questionnaire is 0.80–0.83. The APGAR has been widely used in Chinese families and shows good reliability and validity (Lv, Zeng, Liu, Zhong, & Zhan, 1999). In this study, Cronbach's alpha coefficient for the Family APGAR Index was 0.943.

The Herth Hope Index (HHI) (Herth, 1992) includes 12 items and 3 dimensions: positive attitudes towards reality and the future (4 items), taking positive actions (4 items) and maintaining close relationships with others (4 items). The total score of the scale ranges from 12–48, with scores ranging from 12–23 indicating a low level of hope, 24–35 indicating a medium level and 36–48 indicating a high level. The higher the score of the patient is, the higher the level of hope is. Cronbach's alpha coefficient was 0.87 in this study.

The Acceptance of Illness Scale (AIS) was developed by American scholar Felton, Revenson, & Hinrichsen (1984). This scale is used to measure the degree of acceptance of a disease in adult patients by taking patients with chronic diseases as the research object. The scale contains eight items that are scored using a 5‐point Likert scale ranging from 1 ("strongly agree")–5 ("strongly disagree"). The total score is 8–40 points, and the higher the total score is, the better the patient can accept the discomfort brought on by the disease. A score below 20 indicates low acceptance of the disease, 20–30 moderate acceptance and above 30 high acceptance. Cronbach's alpha coefficient of the scale was 0.849 in this study.

### 3.4 Study design

This multicentre cross‐sectional study used convenience sampling and was conducted at the HD centres of three grade A tertiary hospitals in Sichuan Province, China. The questionnaire and data were kept confidential and used for this study only.

### 3.5 Statistical methods

SPSS 24.0 software was used for data analysis. Measurement data were expressed as x̅ (*SD*); *t* tests and one‐way analysis of variance (ANOVA) were used to test the differences in spiritual health scores of patients with different characteristics. The influencing factors of spiritual health were analysed by using multiple linear regression, where the variable medical expense payment was changed to a dummy variable. Pearson correlation analysis was used to investigate the correlation among the influencing factors. *p* < .05 was considered statistically significant.

### 3.6 Ethical considerations

This study was approved by the Institutional Review Board and complied with the Strengthening the Reporting of Observational Studies in Epidemiology (STROBE) checklist (Appendix S1). Participants were informed of the purpose and significance of the research and signed an informed consent form.

## 4 RESULTS

A total of 423 haemodialysis patients meeting the inclusion criteria were included in this study. After they gave informed consent and completed the questionnaire, 423 questionnaires were recovered, among which 418 were eligible for inclusion, for an effective rate of 98.8%. The demographic data of the patients were obtained, and the average age was 48.07 (*SD* 14.55) years.

### 4.1 Spiritual health scores of MHD patients with different characteristics

The independent‐samples *t* test and one‐way ANOVA were used to test the differences in the spiritual health scores of MHD patients with different characteristics. The results showed that there were statistically significant differences in health scores among people in different ethnic groups, using different medical payment methods and with different religions, hope levels, Family APGAR scores and AIS scores (*p* < .05; Table 1).

**TABLE 1:** Comparison of spiritual health of MHD patients with different characteristics (*N* = 418)

Items	*N*（%）	Score（ $\bar{x}$ ± s）	*t*/*F*	*p*
Age				
18–44	166 (39.7)	30.71 ± 11.87	0.469	.626
45–59	167 (40.0)	30.03 ± 10.60		
≥60	85 (20.3)	29.35 ± 8.55		
Sex				
Male	235 (56.2)	30.72 ± 10.44	1.201	.231
Female	183 (43.8)	29.45 ± 11.11		
Nationality				
Han	373 (89.2)	29.24 ± 10.39	−5.236	<.001
Ethnic minorities	45 (10.8)	37.84 ± 10.65		
Haemodialysis duration (m)				
<24	116 (27.8)	31.22 ± 10.39	1.103	.333
24–60	185 (44.3)	29.36 ± 10.80		
>60	117 (27.9)	30.38 ± 10.98		
Marital status				
Unmarried	82 (19.6)	30.10 ± 10.95	−0.061	.951
Married	336 (80.4)	30.18 ± 10.71		
Educational levels				
Primary school or lower	68 (16.3)	29.03 ± 11.51	0.712	.545
Junior high school	115 (27.5)	30.72 ± 10.91		
Senior high school	103 (24.6)	29.40 ± 10.36		
University or above	132 (31.6)	30.86 ± 10.52		
Personal income (10,00 yuan/month)				
＜2	119 (28.5)	29.87 ± 12.44	1.379	.249
2–4.99	127 (30.4)	31.11 ± 10.18		
5–10	113 (27.0)	28.70 ± 9.81		
＞10	59 (14.1)	31.53 ± 9.79		
Medical payment method				
Urban workers	192 (45.9)	30.43 ± 9.79	5.902	<.001
Urban residents	120 (28.8)	27.29 ± 10.87		
Rural residents	97 (23.2)	32.56 ± 11.65		
Public expense	3 (0.7)	25.10 ± 5.01		
Self‐paying	6 (1.4)	42.83 ± 4.17		
Religious belief				
Yes	57 (13.6)	33.14 ± 11.22	2.263	.024
No	361 (86.4)	29.69 ± 10.61		
Hope level				
Low	0	/	−8.759	<.001
Medium	268 (64.1)	27.50 ± 7.38		
High	150 (35.9)	39.94 ± 12.57		
Family APGAR				
Severely dysfunctional family	56 (13.4)	23.31 ± 5.23	38.435	<.001
Moderately dysfunctional family	128 (30.6)	24.72 ± 6.26		
Functional family	234 (56.0)	34.87 ± 11.14		
Illness acceptance				
Low	108 (25.9)	30.12 ± 11.53	40.173	<.001
Medium	230 (55.0)	29.04 ± 8.45		
High	80 (19.1)	44.15 ± 11.56		

Note

1 yuan = £0.11 sterling

### 4.2 Spiritual health scores of the MHD patients

The total spiritual health score of the MHD patients was 30.16 (*SD* 10.74), and the scores for each dimension were as follows: peace (11.43 *SD* 3.78), meaning (9.92 *SD* 3.56) and faith (8.81 *SD* 3.42; Table 2).

**TABLE 2:** Status of spiritual health of MHD patients (*N* = 418)

Items	Score（ $\bar{x}$ ± S）	Range	Average score ( $\bar{x}$ ± S）
Peace	11.43 ± 3.78	0–16	2.86 ± 0.95
Meaning	9.92 ± 3.56	0–16	2.48 ± 0.89
Faith	8.81 ± 3.42	0–16	2.20 ± 0.86
Total score	30.16 ± 10.74	0–48	2.51 ± 0.90

### 4.3 Correlation between spiritual health and relevant factors in MHD patients

Spearman's correlation analysis revealed that hope level (*r* = .63, *p *< .001), Family APGAR score (*r* = .66, *p *< .001) and AIS score (*r* = 0.37, *p *< .001) were correlated with spiritual health (see Table 3).

**TABLE 3:** Correlation analysis of spiritual health and various factors in MHD patients

Items	*r* (*p*)
Hope level	.63 (<.001)
Family APGAR	.66(<.001)
Illness acceptance	.37 (<.001)

### 4.4 Multiple linear regression analysis of the spiritual health of the MHD patients

The variables with statistical significance in the difference test were taken as independent variables and the spiritual health scores as dependent variables, and multiple linear regression was carried out. Among them, the multicategory discontinuous variables, such as the medical payment method, were converted into dummy variables and the assignment of independent variables is shown in Table 4. The results showed that the independent variables nationality, hope level, Family APGAR score and AIS score all positively predicted the spiritual health scores (*p* < .05; Table 5).

**TABLE 4:** Independent variables in regression analysis of spiritual health‐influencing factors

Independent variables	Items	Assignment of independent variables
X1	Nationality	Han = 1, Ethnic minorities = 2
X2	Urban workers	Urban workers = 0, Urban residents = 1, Rural residents = 0, Public expense = 0, self‐paying = 0
X3	Urban residents	Urban workers = 0, Urban residents = 0, Rural residents = 1, Public expense = 0, self‐paying = 0
X4	Rural residents	Urban workers = 0, Urban residents = 0, Rural residents = 0, Public expense = 1, self‐paying = 0
X5	Public expense	Urban workers = 0, Urban residents = 0, Rural residents = 0, Public expense = 0, self‐paying = 1
X6	Religion	Yes = 1, No = 2
X7	Hope level	Actual scores
X8	Family APGAR	Actual scores
X9	Illness acceptance	Actual scores

**TABLE 5:** Multiple Linear Regression Analysis of Spiritual Health of MHD Patients（*N* = 418）

Items	*B*‐value	*SE*	*β*‐value	*t*‐value	*p*‐value
Constant	−16.842	3.238		−5.201	<.001
Nationality	4.074	1.419	0.130	2.871	.004
Hope level	9.369	1.089	0.397	8.604	<.001
Family APGAR	5.655	0.841	0.307	6.728	<.001
Illness acceptance	3.786	0.819	0.207	4.624	<.001

Note

*R* ^2 ^= .439, *F* = 57.202, *p*<.001.

## 5 DISCUSSION

The results of this study showed that the total spiritual health score of the MHD patients was 30.16 (*SD* 10.74), indicating a moderate level of spiritual health; it was lower than the results of studies performed in other countries (Alradaydeh & Khalil, 2018; Musa, Pevalin, & Al Khalaileh, 2018; Okhli, Hojjati, Sadeghloo, Molaei, & Shahrabady, 2019). The dimension with the highest score was peace, indicating that the MHD patients in this study were relatively calm and able to comfort themselves and feel inner harmony; the dimension with the lowest score was faith, perhaps because only 13.6% of the MHD patients in this study had religious beliefs. In Chinese tradition, spirituality is primarily associated with religion; thus, most patients could not gain mental support from religious beliefs and lost hope and confidence regarding the disease, leading to the faith score being the lowest. However, spirituality extends beyond religion to include everything from social work to education to psychology. Therefore, medical staff should help patients gain a correct understanding of spirituality to improve their spiritual health.

In this study, the results showed that the spiritual health of ethnic minorities was better than that of Han participants and the difference was statistically significant (*p* < .05). China, with 56 ethnic groups, is a multiethnic country with different customs and cultures. At present, there has been no in‐depth research on the spiritual health of Chinese minorities receiving MHD, but according to the existing research, several scholars have found that the mental health of ethnic minority people is better than that of Han people (Su, Li, Wang, & Wang, 2015). Furthermore, good mental health has been shown to be positively correlated with good spiritual health (Lissoni, 2008), consistent with the results of this study. The reasons may be related to the unique national culture and lifestyle of ethnic minorities. China's ethnic minorities are good at singing and dancing and there are festivals almost every month of the year, which increases the channels by which patients can communicate with the outside world and helps resolve psychological problems. In contrast, the spiritual and cultural life of Han people is less vibrant and there are fewer channels for resolving psychological problems. It is suggested that medical staff should pay more attention to Han patients in the future, determine the spiritual distress of patients and meet their spiritual health‐related needs to improve their spiritual health and quality of life.

The results of this study showed that the hope level was an influencing factor of the spiritual health of haemodialysis patients and was positively correlated with spiritual health. The higher the hope level was, the better the spiritual health of the patients was, and the difference was statistically significant (*p* < .05), which is consistent with a previous study (Tavassoli, Darvishpour, Mansour‐Ghanaei, & Atrkarroushan, 2019). Hope is a state associated with positive expectations, an important regulatory mechanism for chronic disease and a powerful multidimensional and underlying factor for recovery and effective adaptation. In other studies, it tends to be referred to as a factor that predicts the development of severe disease (Baljani, Kazemi, Amanpour, & Tizfahm, 2011). The changes in patients' daily lives caused by dialysis can easily cause them to lose hope. If dialysis patients have hope for the future, they will feel better about their quality of life in different respects and having an ideal life can also increase their sense of hope (Fouladi, Ebrahimi, Manshaei, Afshar, & Fouladi, 2014). Raising the level of hope is an effective way to improve the quality of life of patients with chronic diseases. The enhancement of hope can improve the spiritual health and quality of life of patients.

The results showed that family function was a positive predictor of the spiritual health of MHD patients, and the better the family function, the better the spiritual health. Family is the core of society and provides not only material support but also spiritual and emotional support. Studies have shown that good family support improves the spiritual health of patients (Spinale et al., 2008), which is consistent with our study. Family function plays a crucial role in the growth of individuals. If the family does not perform its basic functions in its operation, family members will have a variety of problems. It is believed that the level of family function is a reflection of a family's ability to meet the needs of its members and good family care can effectively relieve or alleviate the psychological symptoms of patients, which is helpful for the treatment of haemodialysis patients (Cicolini, Palma, Simonetta, & Di Nicola, 2012). However, with the prolongation of the course of the disease and time on dialysis of haemodialysis patients, family members will experience burnout; this may affect the degree of family care and even cause conflict or intensify conflicts between family members, cooling family relations (Çelik, Annagur, Yılmaz, Demir, & Kara, 2012). This suggests that medical staff should inform the family members to further strengthen the care provided for and communication with the patient and improve the patient's family function, which can help the patients establish a good attitude and improve their spiritual health.

In this study, the results showed that patients with higher disease acceptance had better spiritual health and the difference was statistically significant (*p* < .05). Acceptance of illness means that the patient understands the purpose of haemodialysis, can tolerate various complications during the treatment process and can better adapt to the changes brought on by the disease, thereby improving the patient's health and satisfaction with life (Jankowska‐Polańska et al., 2017). A previous study showed that disease acceptance in haemodialysis patients can predict better health (Łatka, Majda, & Sołtys, 2013). The higher the degree of disease acceptance, the better the quality of life, the better the physical function and emotional state, the higher the subjective well‐being and life satisfaction and the higher the treatment compliance. At present, there are few intervention studies on the disease acceptance of haemodialysis patients, so medical staff should adopt targeted intervention measures according to the influencing factor of disease acceptance to improve patients' disease acceptance and thereby their spiritual health.

## 6 CONCLUSION

In conclusion, the MHD patients exhibited a moderate level of spiritual health. Multiple linear regression analysis suggested that nationality, hope, family function and acceptance of illness were significant predictors of spiritual health. Patients who have higher hope levels, better family support and better illness acceptance may maintain better spiritual health. These findings can help medical staff identify influencing factors and improve the spiritual health of MHD patients.

### 6.1 Relevance to clinical practice

Spiritual health is a type of health that transcends physical, mental and social levels and is called the fourth dimension of health by the WHO, which identified it as an important factor affecting the quality of life of haemodialysis patients. Spiritual health enables people to have strong faith and hope to constantly transcend adversity and achieve their life goals. Patients with good spiritual health have a higher quality of life. In China, studies of spiritual health have mainly concentrated on cancer patients, with few reports on the study of MHD patients. Therefore, it is important to consider the spiritual health of MHD patients in China and adopt targeted nursing strategies to improve their spiritual health.

### 6.2 Limitations

The limitation of this study is that only three hospitals in Sichuan Province were investigated, which may affect the representativeness of the samples, and the sample size needs to be expanded in the future to investigate the spiritual health of patients from different regions. In addition, qualitative research can be carried out to further understand the factors affecting patients' spiritual health to provide a reliable reference for spiritual health intervention and construct spiritual care models.

## CONFLICT OF INTEREST

The authors declare that they have no conflict of interest.

## Supporting information

AppendixS1
